# Reactive Arthritis Associated With Helicobacter Pylori Infection Presenting as Treatment-Refractory Monoarthritis: A Case Report

**DOI:** 10.7759/cureus.109533

**Published:** 2026-05-24

**Authors:** Yarden Assabag

**Affiliations:** 1 Rheumatology, Clalit Health Services, Beer Sheva, ISR

**Keywords:** clinical reasoning, extra-gastric manifestations, helicobacter pylori, h. pylori eradication, reactive arthritis, seronegative monoarthritis

## Abstract

*Helicobacter pylori* (*H. pylori*) is a prevalent gastric pathogen increasingly recognized as a potential trigger for extra-gastric inflammatory conditions. Despite this, its association with reactive arthritis (ReA) remains under-reported and is often overlooked in the differential diagnosis of seronegative arthritis. A 19-year-old female presented with a five-month history of persistent inflammatory monoarthritis of the left knee. The condition was refractory to non-steroidal anti-inflammatory drugs (NSAIDs), intra-articular corticosteroid injections, and conventional synthetic disease-modifying antirheumatic drugs (csDMARDs), including methotrexate and sulfasalazine. Clinical investigations revealed an inflammatory, aseptic synovial effusion, while serological markers - including antinuclear antibodies (ANA), rheumatoid factor (RF), and anticyclic citrullinated peptide (anti-CCP) - and human leukocyte antigen B27 (HLA-B27) were negative. A concurrent evaluation for dyspepsia identified an active *H. pylori *infection via a urea breath test. Following the discontinuation of all immunosuppressive therapy and the administration of a 10-day triple eradication regimen, the patient achieved complete and sustained clinical and biochemical remission, which was maintained at a three-month follow-up. This case underscores the importance of considering *H. pylori *as a causative agent in cases of unexplained, treatment-refractory monoarthritis. Systematic screening for *H. pylori *may identify a reversible infectious trigger, potentially preventing unnecessary long-term immunosuppression and biological therapy in young patients.

## Introduction

Reactive arthritis (ReA) is an inflammatory arthropathy that develops following infection at a distant site, most commonly in the gastrointestinal or genitourinary tract. Classical triggering pathogens include *Chlamydia trachomatis*, *Salmonella*, *Shigella*, *Campylobacter*, and *Yersinia *species [1]. The condition predominantly affects young adults and typically presents as an asymmetric oligoarthritis of the lower extremities. While the precise pathogenesis remains incompletely understood, molecular mimicry and cross-reactive immune responses between microbial antigens and host joint tissues are considered central mechanisms [2].

*Helicobacter pylori *(*H. pylori*) is a gram-negative spiral bacterium that colonizes the gastric mucosa of approximately 50% of the global population [3]. It is the primary causative agent of chronic gastritis and peptic ulcer disease and is classified as a Group 1 carcinogen for gastric adenocarcinoma. Beyond its gastric manifestations, *H. pylori *has been implicated in a range of extra-digestive conditions, encompassing cardiovascular, hematological, dermatological, and neurological disorders [4]. Its potential role in inflammatory joint disease, however, remains a relatively unexplored area.

The association between *H. pylori* infection and musculoskeletal inflammation has been described in scattered case reports and small case series over the past two decades [5,6]. Proposed pathogenic mechanisms include molecular mimicry between *H. pylori*-derived antigens, particularly heat shock protein 60 (HSP60) and outer membrane proteins (OMPs), and human cartilage or joint tissue components. Additionally, chronic systemic immune dysregulation driven by persistent gastric colonization may play a key role [6,7]. Despite these observations, *H. pylori* is not routinely considered in the differential diagnosis of seronegative inflammatory arthritis, and screening is infrequently performed in this clinical context.

We present a case of treatment-refractory inflammatory monoarthritis in a young woman that resolved completely and durably following *H. pylori* eradication therapy. This case adds to the limited evidence supporting this association and underscores the potential clinical relevance of infectious triggers in unexplained seronegative arthritis cases.

## Case presentation

A 19-year-old woman first presented to her primary care physician in August 2025 with pain, swelling, and warmth of the left knee, accompanied by prolonged morning stiffness. She denied any preceding diarrheal illness, urinary symptoms, or recent genitourinary infection. She had no personal or family history of psoriasis, inflammatory bowel disease, or axial spondyloarthropathy. She reported a remote history of *H. pylori* infection, diagnosed several years prior, for which she was not treated, as she remained entirely asymptomatic at that time. An intra-articular corticosteroid injection was administered at this initial presentation, with no sustained benefit.

She was subsequently referred to the rheumatology outpatient clinic in September 2025. Physical examination revealed a moderately effused left knee with periarticular warmth and tenderness. Range of motion was restricted due to pain. No rash, nail changes, oral ulcers, ocular inflammation, or FABER (flexion, abduction, and external rotation) test abnormality was identified. Examination of the axial skeleton and peripheral joints was otherwise unremarkable.

Laboratory investigations revealed a marginally elevated C-reactive protein (CRP) of 5 mg/L (reference range: <5 mg/L), consistent with a mild acute-phase response, with a normal erythrocyte sedimentation rate (ESR). Full blood count was within normal limits. Autoimmune serology was comprehensively negative, including antinuclear antibodies (ANA), rheumatoid factor (RF), anticyclic citrullinated peptide (anti-CCP) antibodies, and complement levels. HLA-B27 testing was negative.

Arthrocentesis of the left knee was performed on two separate occasions at the referring center, yielding 20 mL of synovial fluid. Laboratory analysis of the synovial fluid revealed 15,000 white blood cells per microliter, with a predominance of neutrophils. No crystals were identified under polarized light microscopy. Extensive microbiological analysis - including Gram stain, Ziehl-Neelsen stain for mycobacteria, fungal smear, and aerobic/anaerobic bacterial cultures - remained consistently negative across these investigations, confirming the sterile, aseptic nature of the inflammatory effusion. Synovial fluid was not sent for PCR-based pathogen detection or 16S rRNA sequencing, which represents a limitation of the microbiological workup. Magnetic resonance imaging (MRI) of the left knee demonstrated joint effusion and synovitis without erosions, enthesopathy, or cartilage loss, as illustrated in Figure 1.

**Figure 1:**
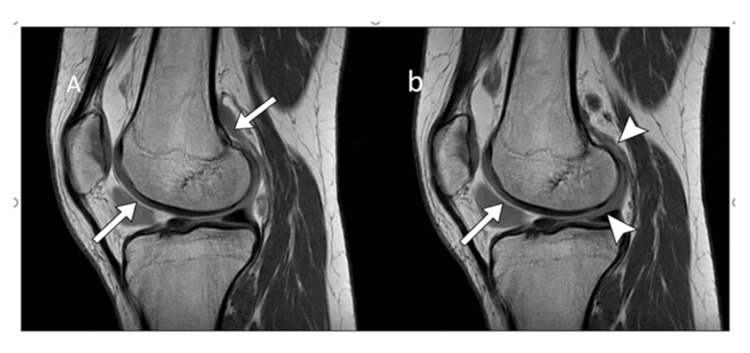
Magnetic resonance imaging (MRI) of the left knee (A) Sagittal view demonstrating significant joint effusion (white arrows) and synovial thickening. (B) Sagittal view highlighting active synovitis (white arrowheads) along the joint capsule. There are no signs of bone erosions, enthesopathy, or cartilage loss.

Given the clinical presentation of inflammatory monoarthritis and concurrent gastrointestinal manifestations, in the absence of HLA-B27, a provisional diagnosis of reactive arthritis was made. The patient was commenced on methotrexate 15 mg weekly and naproxen 500 mg twice daily. In view of an insufficient response after one month, sulfasalazine 1 g twice daily was added to the methotrexate in October 2025, yielding a combination DMARD (disease-modifying antirheumatic drug) regimen. Despite this escalation, no clinical improvement was apparent after a further month of combination therapy.

Coincidentally, during this period, the patient was evaluated by her primary care physician for dyspeptic symptoms comprising epigastric discomfort, postprandial fullness, and nausea. In light of her prior history of *H. pylori* infection, a urea breath test (UBT) was performed, which returned a positive result, indicating active *H. pylori *infection. All antirheumatic therapy was discontinued in November 2025, and standard triple eradication therapy was commenced, comprising amoxicillin 1 g twice daily, clarithromycin 500 mg twice daily, and omeprazole 20 mg twice daily for 10 days.

Within several weeks of completing eradication therapy, the patient reported progressive and marked improvement in left knee pain and morning stiffness. By January 2026, her articular symptoms had resolved completely. Laboratory investigations in December 2025 demonstrated normalization of the CRP (2 mg/L) and ESR (6 mm/h), with persistently negative RF and anti-CCP antibodies.

At the rheumatology follow-up in February 2026, the patient was not receiving any antirheumatic medication and reported complete resolution of knee pain with no recurrence. Physical examination of the left knee revealed no effusion, warmth, or synovitis. Examination was also notable for the absence of rash, nail changes, uveitis, and a normal FABER test bilaterally. A post-eradication UBT was not performed. A summary of the clinical course is presented in Table 1.

**Table 1:** Clinical and therapeutic timeline DMARD: Disease-modifying antirheumatic drug.

Date	Event
Aug-25	Onset of left knee pain, swelling, and morning stiffness; intra-articular corticosteroid injection administered
Sep-25	Arthrocentesis (15,000 WBC/µL, negative cultures); MRI confirmed synovitis
	HLAB27 negative, methotrexate 15 mg/week + naproxen 500
Oct-25	Sulfasalazine 1 g BD added to methotrexate (combination DMARD therapy); no clinical improvement
Nov-25	Positive urea breath test identified; all antirheumatic therapy discontinued; *H. pylori* triple eradication therapy commenced (amoxicillin + clarithromycin + omeprazole x 10 days)
Dec-25	Progressive resolution of knee pain and stiffness; C-reactive protein (CRP) 2 mg/L; erythrocyte sedimentation rate (ESR) 6 mm/h
Jan-26	Complete resolution of articular symptoms; off all medication
Feb-26	Rheumatology review: no synovitis, no arthritis recurrence, off all medication

## Discussion

We report a case of treatment-refractory seronegative monoarthritis in a young, HLA-B27-negative woman that resolved completely and durably following *H. pylori *eradication therapy. Over a four-month disease course, the arthritis failed to respond to intra-articular corticosteroid injection, NSAIDs, and combination DMARD therapy with methotrexate and sulfasalazine before achieving complete remission within weeks of eradication therapy. To our knowledge, this represents one of the few published cases documenting this association in the context of failed conventional disease-modifying therapy.

Reactive arthritis associated with *H. pylori *has been described in the literature since the 1990s, though reports remain sparse. Current evidence supports two principal non-exclusive pathways: molecular mimicry between *H. pylori*-derived antigens, particularly heat shock protein 60, OMPs, and urease, and human cartilage components; and chronic systemic immune dysregulation driven by persistent gastric colonization [6,7].

It is important to distinguish *H. pylori*-associated ReA from septic arthritis. ReA is, by definition, an aseptic inflammatory process. The absence of cultivable pathogens in the joint fluid, as demonstrated in our case, is a hallmark of the condition and supports a systemic immune-mediated mechanism rather than a local infectious process.

Several features of this case warrant specific discussion. First, the negative HLA-B27 status is consistent with the majority of published *H. pylori*-associated reactive arthritis cases, suggesting a pathogenic mechanism distinct from classical enteric-triggered spondyloarthropathy - one perhaps more consistent with the non-HLA-restricted extra-gastric autoimmune manifestations of *H. pylori *infection. An alternative explanation is that this case represented undifferentiated spondyloarthropathy coincidentally coexisting with *H. pylori *infection and undergoing spontaneous remission; however, this possibility cannot be entirely excluded. However, the failure of combination DMARD therapy and the tight temporal relationship between eradication and remission support a causal rather than incidental association.

Second, the treatment-refractory course is clinically significant. The sequential failure of four therapeutic interventions, including combination methotrexate and sulfasalazine, over four months argues strongly against spontaneous remission. The temporal relationship between eradication therapy and symptom resolution, with complete remission occurring within weeks and sustained for three months off all therapy, provides the strongest available evidence for a causal association.

Although methotrexate and sulfasalazine were initiated, the rapid resolution of symptoms following *H. pylori *eradication suggests a primary role of the infection in the inflammatory process, as these medications typically require a longer therapeutic lead time.

Third, the route by which *H. pylori *was identified underscores a recognized gap in clinical practice. If the infectious trigger had not been identified coincidentally by the primary care physician, the patient might have been unnecessarily exposed to biologic agents.

The association between *H. pylori *and reactive arthritis is supported by several case reports [8]. While the exact immunologic mechanism remains to be fully elucidated, the temporal relationship observed, where clinical resolution followed *H. pylori *eradication, aligns with existing literature [8]. However, given the observational nature of these reports, we present these findings as a temporal association rather than definitive causation.

Several limitations should be acknowledged.A post-eradication UBT was not performed, which prevents definitive microbiological confirmation of eradication. However, the complete and sustained resolution of both gastrointestinal and articular symptoms in the absence of any antirheumatic therapy is temporally consistent with successful eradication-driven remission. Additionally, synovial fluid was not analyzed by PCR-based or 16S rRNA sequencing methods, which represents a limitation of the microbiological exclusion. While the follow-up period is currently three months following remission, the complete absence of recurrence while off all immunosuppressive therapy strongly supports the diagnosis.

## Conclusions

*H. pylori* represents an underrecognized yet potentially significant trigger of seronegative inflammatory arthritis. While a definitive causal relationship cannot be established on the basis of this observational report, our findings underscore the clinical utility of incorporating *H. pylori *screening into the diagnostic workup of monoarticular inflammatory arthritis, particularly in cases refractory to conventional DMARD therapy. Further studies are warranted to elucidate the underlying immunopathogenic mechanisms and to define the broader clinical implications of this association.
